# High production of enantiopure (*R,R*)-2,3-butanediol from crude glycerol by *Klebsiella pneumoniae* with an engineered oxidative pathway and a two-stage agitation strategy

**DOI:** 10.1186/s12934-024-02480-4

**Published:** 2024-07-23

**Authors:** Min-Ho Jo, Jung-Hyun Ju, Sun-Yeon Heo, Chang-Bum Son, Ki Jun Jeong, Baek-Rock Oh

**Affiliations:** 1 Microbial Biotechnology Research Center, Jeonbuk Branch Institute, Korea Research Institute of Bioscience and Biotechnology (KRIBB), Jeongeup, Jeonbuk 56212 Republic of Korea; 2 Department of Chemical and Biomolecular Engineering and Institute for the BioCentury, KAIST, Daejeon, 34141 Republic of Korea

**Keywords:** (*R,R*)-2,3-butanediol, *Klebsiella pneumoniae*, Crude glycerol, Oxidative pathway, Two-stage agitation strategy

## Abstract

**Background:**

(*R,R*)-2,3-butanediol (BDO) is employed in a variety of applications and is gaining prominence due to its unique physicochemical features. The use of glycerol as a carbon source for 2,3-BDO production in *Klebsiella pneumoniae* has been limited, since 1,3-propanediol (PDO) is generated during glycerol fermentation.

**Results:**

In this study, the inactivation of the *budC* gene in *K. pneumoniae* increased the production rate of (*R,R*)-2,3-BDO from 21.92 ± 2.10 to 92.05 ± 1.20%. The major isomer form of *K. pneumoniae* (*meso*-2,3-BDO) was shifted to (*R,R*)-2,3-BDO. The purity of (*R,R*)-2,3-BDO was examined by agitation speed, and 98.54% of (*R,R*)-2,3-BDO was obtained at 500 rpm. However, as the cultivation period got longer, the purity of (*R,R*)-2,3-BDO declined. For this problem, a two-step agitation speed control strategy (adjusted from 500 to 400 rpm after 24 h) and over-expression of the *dhaD* gene involved in (*R,R*)-2,3-BDO biosynthesis were used. Nevertheless, the purity of (*R,R*)-2,3-BDO still gradually decreased over time. Finally, when pure glycerol was replaced with crude glycerol, the titer of 89.47 g/L of (*R,R*)-2,3-BDO (1.69 g/L of *meso*-2,3-BDO), productivity of 1.24 g/L/h, and yield of 0.35 g/g consumed crude glycerol was achieved while maintaining a purity of 98% or higher.

**Conclusions:**

This study is meaningful in that it demonstrated the highest production and productivity among studies in that produced (*R,R*)-2,3-BDO with a high purity in *Klebsiella* sp. strains. In addition, to the best of our knowledge, this is the first study to produce (*R,R*)-2,3-BDO using glycerol as the sole carbon source.

**Supplementary Information:**

The online version contains supplementary material available at 10.1186/s12934-024-02480-4.

## Background

As growing concerns and awareness of environmental issues, the world is shifting away from petroleum-based chemical manufacturing and toward biorefinery, which enables carbon–neutral production by using biomass, a sustainable feedstock [1, 2]. Glucose, which generally originates from starch, has traditionally been utilized in biorefinery. However, it is associated with a lower profit margin in biorefinery due to its relatively high cost, as well as there are also issues regarding the usage of a food resource [3]. Thus, many researchers have investigated the development of novel biorefinery techniques that use industrial waste or non-food resources [4]. Among them, glycerol is a noteworthy non-food resource and low-cost feedstock for a sustainable and circular bioeconomy [5].

Glycerol is a by-product of the biodiesel production process [6]. The growing demand for biodiesel is going to boost the amount of glycerol available on the market. The disposal of this surplus crude glycerol incurs enormous expenses [7], but microbial conversion to worthwhile compounds using crude glycerol offers a viable alternative [8]. The cost competitiveness of crude glycerol as a feedstock for glycerol-biorefinery is likely to rise as the biodiesel market increases in size. Also, since biorefinery is based on the amalgamation of biomass conversion processes to generate power, fuels, and chemicals, utilizing crude glycerol generated during biodiesel production as a biomass raw material for biorefinery to produce valuable chemicals via microbial fermentation will provide excellent virtuous cycle opportunities [1].

**Fig. 1:**
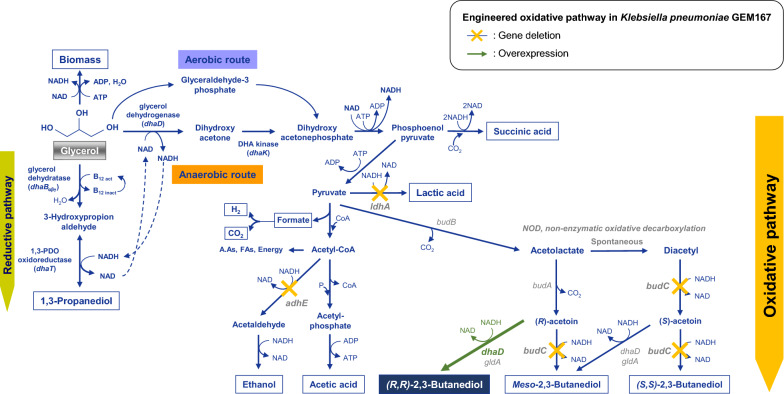
(*R,R*)*-*2,3-BDO biosynthetic pathway from glycerol in *K. pneumoniae* GEM167 *ΔadhEΔldhAΔldhA* with an engineered oxidative pathway

2,3-Butanediol (2,3-BDO) can exists in three stereoisomers [*meso*-, (*R,R*)-, and (*S,S*)-forms]. In the 2,3-BDO biosynthetic pathway of *Klebsiella pneumoniae* (Fig. 1), pyruvate is converted to acetolacate by acetolactate synthase (*budB*). It is then converted to (*R*)-acetoin by acetolactate decarboxylase (*budA*). (*R*)-acetoin is converted to *meso*-2,3-BDO by *meso*-2,3-butanediol dehydrogenase (*budC*) [9]. The isomer form of 2,3-butanediol produced by *K. pneumoniae* and *K. oxytoca* is typically *Meso*-2,3-BDO, and (*S,S*)-2,3-BDO is also partially produced [10]. However, a few studies have indicated that *K. pneumoniae* is able to produces (*R,R*)-2,3-BDO as well as *meso*-2,3-BDO and (*S,S*)-2,3-BDO [11]. Butanediol dehydrogenase is involved in the reversible conversion between acetoin and 2,3-BDO. The specificity of butanediol dehydrogenase determines the stereoisomer of 2,3-BDO. A variety of butanediol dehydrogenases have been identified and characterized. Some butanediol dehydrogenases function in similar role even though they are not annotated as butanediol dehydrogenase [12]. In this regard, it has been reported that *dhaD* and *gldA*, which encode glycerol dehydrogenase, were also involved in (*R,R*)-2,3-BDO biosynthesis [13], and *dhaD* and *gldA* genes were used to enhance the production of (*R,R*)-2,3-BDO [14]. The physicochemical properties of the isomer of 2,3-BDO differ based on the isomer form [10]. First, optically active (*R,R*)-2,3-BDO and (*S,S*)-2,3-BDO have higher structural stability than optically inactive *meso*-2,3-BDO [15]. These optically active 2,3-BDOs are invaluable in applications that require chiral groups such as drugs, high-value pharmaceuticals, and liquid crystals [16, 17]. And (*R,R*)-2,3-BDO and *meso*-2,3-BDO have similar applications in chemical industries such as 1,3-butanediol, methyl ethyl ketone (MEK), printing inks, spandex, and softening agents [15, 18, 19]. On the other hand, (*R,R*)-2,3-BDO has a lower freezing point than *meso*-2,3-BDO, making it suitable for transportation and storage at low temperatures. Given this characteristic, it can also be employed as an antifreeze agent [10, 20]. Among the various applications, (*R,R*)-2,3-BDO has drawn a lot of attention in the agriculture field because of its distinctive physicochemical features that activate plant self-defense systems in response to external stimuli [21].

*K. pneumoniae*, an industrially advantageous microbe, can grow on glycerol as the sole carbon source, and is mainly used to produce 1,3-propanediol (1,3-PDO) through glycerol fermentation [22]. On the other hand, 2,3-butanediol (2,3-BDO) from *K. pneumoniae* has traditionally been produced using glucose rather than glycerol [23]. This is because 1,3-PDO is unavoidably generated as the main byproduct during glycerol fermentation for 2,3-BDO production in *K. pneumoniae*, which complicates the separation process in the downstream process [24]. Thus, it has been restricted to the use of glycerol as a carbon source in the production of 2,3-BDO using *K. pneumoniae.* To overcome the inevitable 1,3-PDO production in glycerol fermentation of *K. pneumoniae* strain, a previous study reported that the *K. pneumoniae* GEM167 mutant strain was used to produce a metabolite derived from the oxidative pathway without the production of 1,3-PDO [25]. The *K. pneumoniae* GEM167 mutant strain has the characteristics of a unique metabolic pathway, with an inhibited metabolite of the reductive pathway (1,3-PDO) and an enhanced metabolites of the oxidative pathway (2,3-BDO, ethanol, lactic acid, and succinate) [26].

In addition, for the above reasons, the majority of biomass feedstock in the study for stereospecific production of (*R,R*)-2,3-BDO has also been centered on glucose, and strains that naturally produce (*R,R*)-2,3-BDO as the main isomer form have been employed [2]. In the case of *Klebsiella* sp., little study has been done to produce (*R,R*)-2,3-BDO, and even these studies used glucose as a carbon source [10, 14]. These studies are as follows; *K. oxytoca ΔldhAΔpflBΔbudC*::PBDH (pBBR-PBDH) produced 92% of (*R,R*)-2,3-BDO at 106.7 g/L (*meso*-2,3-BDO at 9.3 g/L) [10], and *K. pneumoniae ΔwabGΔbudCΔldhA*::*gldA*::*dhaD* produced 98% of (*R,R*)-2,3-BDO at 61 g/L (*meso*-2,3-BDO at 1.4 g/L) [14]. And no studies have been reported to produce (*R,R*)-2,3-BDO using glycerol in *Klebsiella* sp..

Therefore, this study aimed to produce enantiopure (*R,R*)-2,3-BDO by metabolic engineering and culture process factor (agitation speed) in *K. pneumoniae* GEM167 mutant strain from glycerol, a byproduct of biodiesel, as a sole carbon source.

## Materials and methods

### Development of strains and culture media

Table 1 lists the bacterial strains employed in this study. DNA manipulation was carried out with *Escherichia coli* DH5α. As helper plasmids, pKD46 [27] and pCP20 [28] were used to express Lambda-Red recombinases and Flippase (FLP) recombinases, respectively. Replication of these plasmids makes them easier to eliminate because of their temperature sensitivity. The apramycin-resistant gene was supplied via the pIJ773 vector. Cloning was conducted using the pGEM-T Easy vector (Promega Co., USA) and the pBHA vector (Bioneer Co., Korea).

**Table 1:** Bacterial strains used in this study

Strains or plasmids	Genotype and description	Source
Strains		
*E. coli* DH5α	Host of plasmid	Lab stock
*K. pneumoniae* GEM-adhE-ldhA	*K. pneumoniae ΔadhEΔldhA*	This study
*K. pneumoniae* GEM-adhE-ldhA-budC	*K. pneumoniae ΔadhEΔldhAΔbudC*	This study
Plasmids		
pETM6-T7-Amp.^R^	T7 promoter, pETM6, Amp.^R^	Bioneer Co. (Korea)
pETM6-T7-dhaD-Tet.^R^	T7 promoter, pETM6 carrying *dhaD*, Tet.^R^	This study

*Amp.* Ampicillin, *Tet.* tetracycline

Bacterial strains were grown in either Luria–Bertani (LB) medium (LB-broth Miller, Formedium, Hunstanton, UK) or LB medium supplemented with antibiotics (ampicillin [100 μg/mL] and/or apramycin [50 μg/mL]) were used. Then, the flask medium for seed culture and 5L-fermentor medium were prepared and used in the same manner as in the previous experiments [26].

### Gene knock-out for metabolic flux shift

Figure S1 illustrates the strategy for knocking out the gene for acetoin reductase (*budC*), which catalyzes the formation of 2,3-butanediol from acetoin [29] (see Additional file 1). Table S1 provided the oligonucleotides used for PCR amplification of the gene’s upstream and downstream regions.

The amplified fragments were fused with primers P1 and P4, which were subsequently introduced into the pGEM-T Easy vector. The Klenow fragment-processed apramycin-resistance gene (*aac(3)IV*) was inserted into the PCR product. The built plasmids, such as pT-*budC*-*Apra* functioned as the deletion cassette [29]. It was then introduced into *K. pneumoniae* GEM167* ΔadhEΔldhA* using the electroporation method [30], and homologous recombination yielded chromosomal variants. Finally, it was demonstrated whether DNA fragments were integrated by homologous recombination using primers P5 and P6 (Additional file 1: Table S1).

### Construction of recombinant plasmid

Figure S2 depicted the strategy used to construct plasmid that increased the expression of *dhaD* (encoded in glycerol dehydrogenase, KPN2242_20560 from *K. pneumoniae*) gene, which has a dual function in glycerol metabolism and 2,3-butanediol formation. The gene was synthesized by Bioneer Co. Ltd. (Daejeon, South Korea). The sequence was cloned into the pBHA vector and followed by sequencing to ensure there were no errors. And then, the DNA fragment of *dhaD* gene was ligated to pETM6-T7-Amp.^R^ by insertion of *Nde*I-*Xho*I fragments containing *dhaD* (pBHA-dhaD) into the *Nde*I-*Xho*I site of pETM6-T7-Amp.^R^. Next, to replace ampicillin-resistance gene with tetracycline- resistance gene, the *Xho*I fragment with treatment of the Klenow fragment (pGEM-T Easy vector carrying tetracycline-resistance gene) that contained tetracycline-resistance gene was inserted into the *Sca*I site of ampicillin-resistance gene, resulting in pETM6-T7-dhaD-Tet.^R^. Electroporation was employed to introduce the final plasmid into *K. pneumoniae* [31].

### Fermentation by *K. pneumoniae* strains

The culture conditions for seed cells were 37 °C and 200 rpm. Seed cells were cultivated in LB medium for 9 h. These were inoculated into flask medium (30 g/L glycerol, 1 g/L yeast extract, 2 g/L (NH_4_)_2_SO_4_, 10.7 g/L K_2_HPO_4_, and 5.24 g/L KH_2_PO_4_) and cultured for 12 h. It was then inoculated into a 5 L jar-fermentor (CNS Co., Ltd, Korea) with 10% (v/v) inoculum. 5L-fermentor medium contained 20 g/L glycerol, 1 g/L yeast extract, 2 g/L (NH_4_)_2_SO_4_, 10.7 g/L K_2_HPO_4_, and 5.24 g/L KH_2_PO_4_. The following compounds were added to all flask medium and 5 L-fermentor medium: 0.2 g/L MgSO_4_, 0.02 g/L CaCl_2_·2H_2_O, 1 mL Fe solution (5 g/L FeSO_4_·7H_2_O and 4 mL HCl [37%, w/v]), 1 mL trace element solution (70 mg/L ZnCl_2_, 100 mg/L MnCl_2_·4H_2_O, 60 mg/L H_3_BO_3_, 200 mg/L CoCl_2_·4H_2_O, 20 mg/L CuCl_2_·2H_2_O, 25 mg/L NiCl_2_·6H_2_O, 35 mg/L Na_2_MoO_4_·2H_2_O, and 4 mL HCl [37%, w/v]), or 10 μg/mL tetracycline. Fed-batch fermentation was carried out in 2.5 L of fermentor medium (37 ℃, 700 rpm, and aeration at 2.0 vvm) [25]. The pH was controlled through the automated addition of ammonia solution. The pure glycerol (99%, w/w) [29] or crude glycerol (80%, w/w) obtained from GS Bio (Yeosu, Korea) were used as a carbon source. Isopropyl β-D-1-thiogalactopyranoside (IPTG) was added to the culture medium as an inducer (final concentration: 0.5 mM).

### RNA extraction, reverse transcription, and real-time reverse transcription PCR (real-time RT-PCR)

Total RNA from samples at 9 h of culture of each *K. pneumoniae* strain was extracted using the TaKaRA MiniBEST Universal RNA Extraction kit (Takara Bio Inc., Japan).

PrimeScript™ reverse transcriptase (Takara, Japan) was employed to synthesize the complementary DNA from each RNA sample. First, a mixture of extracted RNA, Oligo (dT) primer, dNTP, and RNase free water was incubated at 65 ℃ for 5 min. Then, PrimeScript^™^ reverse transcriptase, 5 × PrimeScript buffer, and RNase inhibitor were added to the aforementioned solution and incubated at 37 ℃ for 15 min. Lastly, the mixture was heated at 85 ℃ for 5 s and cooled on ice. The resulting reactant served as the template for the real-time RT-PCR experiment. PrimeScript^™^ RT Master Mix (Takara, Japan) and a qTOWER^3^ from AnalytikJena (Jena, Germany) were used for the real-time RT-PCR experiment and for ΔΔCt analysis. The primers used in the real-time RT-PCR experiment are provided in Table S2 [14].

To normalization the results of gene expression from each experiment, *rpoD* (encoded in RNA polymerase sigma factor) from *K. pneumoniae* was used as a reference gene. To determine the relative gene expression level, the fold-change value was calculated according to the 2^−ΔΔCT^ method using measured Ct (threshold cycle) value [32].

### Analytical methods

To determine cell growth, optical density (O.D) was measured at a wavelength of 600 nm. A high-performance liquid chromatography (HPLC) analysis based on prior research methods [33] was performed to quantify the concentrations of metabolites (glycerol, lactic acid, acetic acid, succinic acid, 1,3-PDO, and ethanol) in fed-batch fermentation.

A gas chromatography (GC) system (Agilent Technologies 6890N, Agilent Technologies, Santa Clara, CA, USA) equipped with a flame ionized detector (FID) and an HP-CHIRAL-20B column (30 m × 0.25 mm × 0.25 μm) was employed to quantify (*S*)-acetoin, (*R*)-acetoin, (*S,S*)-2,3-butanediol, (*R,R*)-2,3-butanediol, and *meso*-2,3-butanediol. Nitrogen was used as the carrier gas with a flow rate of 1.0 mL/min, and the injected volume was 1 μL with a split injection mode (split ratio of 25:1). The injector and the flame ionization detector temperatures were 240 ℃ and 250 ℃, respectively. The gradient program used for controlling the column temperature was as follows: start at 50 ℃ for 1 min, increased at a rate of 10 ℃/min to 80 ℃, isotherm at 80 ℃ for 5 min, increased at a rate of 5 ℃/min to 100 ℃, isotherm at 100 ℃ for 7 min, increase at a rate of 40 ℃/min to 240 ℃, and then maintained at 240 ℃ for 5 min [14].

## Results & discussion

### Shift toward the biosynthetic pathway of *(R,R)*-2,3-butanediol through regulation of gene transcription level by knock-out of *budC* gene

To specifically synthesize (*R,R*)-2,3-BDO using glycerol as a sole carbon source in *K. pneumoniae ΔadhEΔldhA* strain, the *budC* gene involved in *meso*-2,3-BDO biosynthesis was knocked out and shifted to the (*R,R*)-2,3-BDO biosynthesis pathway, as illustrated in Fig. 1. Cultivations were carried out for 24 h at 37℃, 700 rpm, 2.0 vvm, and pH 6 control (using ammonium water).

**Fig. 2:**
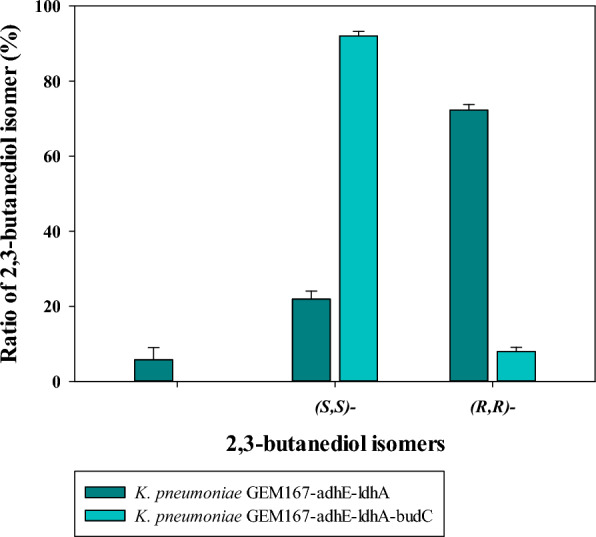
Biosynthetic shift of 2,3-butanediol stereoisomers by knock-out of *budC* gene in *K. pneumoniae* GEM167 *ΔadhEΔldhA*

As shown in Fig. 2 and Fig. S3 (see Additional file 1), *meso*-2,3-BDO was the main isomeric form of 2,3-BDO in *K. pneumoniae ΔadhEΔldhA*, which was present at 72.30 ± 1.50% before the *budC* gene was knocked out. *K. pneumoniae* produced *meso*-2,3-BDO as the primary 2,3-BDO isomer, and minor amounts of (*S,S*)-2,3-BDO, which was consistent with a previous study [10]. The interesting point was that (*R,R*)-2,3-BDO was produced in this study. It is uncommon to report that all three isomeric forms of 2,3-BDO are formed, however, this has been reported in a few *K. pneumoniae* [11].

**Fig. 3:**
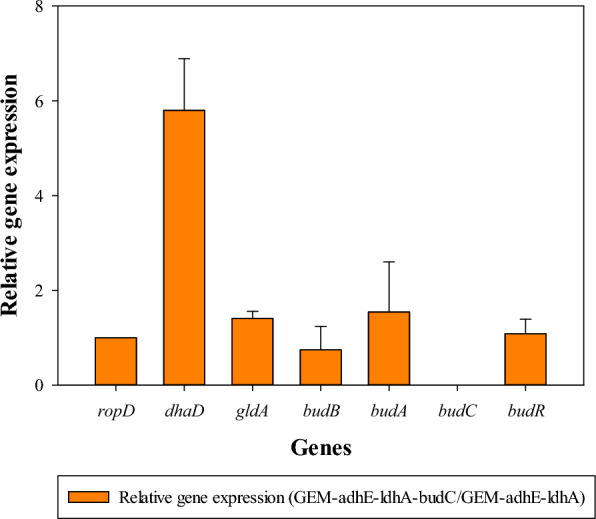
Relative gene expression of *K. pneumoniae ΔadhEΔldhAΔbudC* compared to *K. pneumoniae ΔadhEΔldhA*. The abscissa and ordinate represent strains and relative expression levels of each gene, respectively. Here and below: means ± SDs of triplicate measurements are shown. *SD* standard deviation

Additionally, to investigate the effect on the expression levels of genes associated with the biosynthesis of (*R,R*)-2,3-BDO (*dhaD*, *gldA*, *budB*, *budA*, *budC*, and *budR*) in response to knock-out of the *budC* gene in *K. pneumoniae ΔadhEΔldhA*, the relative gene expression levels of genes related to the biosynthesis of (*R,R*)-2,3-BDO between *K. pneumoniae ΔadhEΔldhA* and *K. pneumoniae ΔadhEΔldhAΔbudC* were compared. As a result Fig. 3, demonstrated that knock-out of *budC* in *K. pneumoniae ΔadhEΔldhAΔbudC* completely prevented expression of *budC* while significantly boosting expression of *dhaD*. Regulating gene expression levels by knocking out of *budC* was as effective as prior study [14], and it is noteworthy that the expression level of *dhaD* increased 5.8-fold in this study. As a result, the production rate of (*R,R*)-2,3-BDO increased from 21.92 ± 2.10 to 92.05 ± 1.20%. The production rate of *meso*-2,3-BDO reduced dramatically from 72.30 ± 1.50 to 7.95 ± 1.15%, and (*S,S*)-2,3-BDO was not detected in GC analysis (Fig. 2). Therefore, in this study, the expression of the gene involved in the 2,3-BDO biosynthesis pathway was regulated by knocking out the *budC* gene, which resulted in the production of (*R,R*)-2,3-BDO as the main isomer of 2,3-BDO by shifting to the (*R,R*)-2,3-BDO biosynthetic pathway. It is the first study to selectively mass-produce (*R,R*)-2,3-BDO using glycerol as a sole carbon source, even if the biosynthetic mechanisms of these 2,3-BDO isomers have been demonstrated through experimental results in previous study [13]. Interestingly, *dhaD* is involved in glycerol catabolism as a glycerol dehydrogenase, and it was expected that glycerol consumption would also increase through the enhanced expression of *dhaD* through knock-out of *budC* [13]. However, in this study, it was observed that glycerol consumption decreased (glycerol consumption of *K. pneumoniae* GEM167-adhE-ldhA and *K. pneumoniae* GEM167-adhE-ldhA-budC was 193.4 ± 9.30 g/L and 171.2 ± 6.45 g/L, respectively) (data was not shown). It suggested that the expressed *dhaD* in this study played a role in the conversion of *R*-acetoin to (*R,R*)-2,3-BDO rather than in glycerol catabolism.

Unfortunately, as indicated in Table 2, the molar conversion of total 2,3-BDO from glycerol consumption was decreased from 0.31 ± 0.01 mol/mol of glycerol to 0.22 ± 0.01 mol/mol of glycerol by the *budC* knock-out. The main reason of these results could be the excessive accumulation of *R*-acetoin caused by the knock-out of *budC* and shift of (*R,R*)-2,3-BDO into the biosynthetic pathway. This was supported by the result that the sum of the molar conversion to *R*- and *S*-acetoin was 0.04 ± 0.01 mol/mol of glycerol, but the molar conversion to *R*-acetoin got doubled to 0.08 ± 0.01 mol/mol of glycerol after *budC* was knocked out. In addition, the increase in molar conversion to acetic acid from 0.03 ± 0.01 mol/mol of glycerol to 0.05 ± 0.01 mol/mol of glycerol was also presumed to have contributed. It has been reported that the biosynthesis of acetoin is closely related to the agitation speed [34, 35]. Therefore, it was considered necessary to investigate the effect of agitation speed on the purity of (*R,R*)-2,3-BDO.

**Table 2:** Molar conversion of metabolites from glycerol in engineered *K. pneumoniae* GEM167 strains after 24 h

Strains	Molar conversion (mol of metabolites/mol of glycerol)											O.D_600_
	Succinate	Lactic acid	Acetic acid	(*R*)-acetoin	(*S*)-acetoin	Total acetoin	(*S,S*)-2,3-BDO	(*R,R*)-2,3-BDO	*Meso*-2,3-BDO	Total 2,3-BDO	Ethanol	
*K. pneumoniae* GEM-adhE-ldhA	0.03 ± 0.00	0.00 ± 0.00	0.03 ± 0.01	0.03 ± 0.01	0.01 ± 0.00	0.04 ± 0.01	0.02 ± 0.01	0.07 ± 0.02	0.22 ± 0.00	0.31 ± 0.01	0.00 ± 0.00	41.60 ± 1.15
*K. pneumoniae* GEM-adhE-ldhA-budC	0.00 ± 0.00	0.00 ± 0.00	0.05 ± 0.01	0.08 ± 0.01	0.00 ± 0.00	0.08 ± 0.01	0.00 ± 0.00	0.20 ± 0.01	0.02 ± 0.00	0.22 ± 0.01	0.00 ± 0.00	42.40 ± 1.52^a^

^a^Fed-batch cultivation was carried out in a 5-L jar fermentor (37 °C, 700 rpm, 2.0 vvm, and pH 6.0 maintained using ammonium water)

### Effect of agitation speed on purity of *(R,R)*-2,3-BDO and two-step agitation speed control strategy

The effect of agitation speed on (*R,R*)-2,3-BDO production and purity was investigated in production of (R,R)-2,3-BDO from glycerol using *K. pneumoniae ΔadhEΔldhAΔbudC* strain. Culture conditions were set according to the agitation speed (400, 500, 600, and 700 rpm) for 24 h based on 37 ℃, 2.0 vvm, and pH 6 adjustment (using ammonia water).

As a result, as shown in Table 3, it was determined that (*R,R*)-2,3-BDO was produced with the highest purity of 98.45 ± 0.10% at an agitation speed of 500 rpm. Also, the highest molar conversion to (*R,R*)-2,3-BDO was 0.33 ± 0.02 mol/mol of glycerol at an agitation speed of 500 rpm in Table S3 (see Additional file 1). Therefore, 500 rpm was determined as the suitable agitation speed to produce high purity of (*R,R*)-2,3-BDO.

**Table 3:** Effects of agitation speed on 2,3-butanediol biosynthesis in *K. pneumoniae* GEM167 *ΔadhEΔldhAΔbudC* after 24 h

Agitation speed (rpm)	Metabolites (g/L)					Purity of (*R,R*)-2,3-BDO (%)
	(*R*)-acetoin	(*S*)-acetoin	2,3-Butanediol isomers			
			(*S,S*)-	(*R,R*)-	*Meso*-	
400	1.04 ± 0.64	–	–	19.97 ± 1.74	0.36 ± 0.08	98.23 ± 0.24
500	2.29 ± 0.77	–	–	32.19 ± 2.73	0.51 ± 0.07	98.45 ± 0.10
600	7.38 ± 0.93	–	–	40.25 ± 3.14	1.23 ± 0.26	97.03 ± 0.39
700	10.47 ± 1.31	–	–	38.60 ± 1.32	2.07 ± 0.20	94.91 ± 0.30^a^

-, not detected

^a^Fed-batch cultivation was carried out in a 5-L jar fermentor (37 °C, 700 rpm, 2.0 vvm, and pH 6.0 maintained using ammonium water) according to the agitation speed (400, 500, 600, and 700 rpm)

It was observed that an increase in agitation speed resulted in an increase in cell growth (Table S3). It was suggested that the increase in agitation speed effectively increased the oxygen transfer efficiency [36], which has a positive effect on cell growth. These results were consistent with previous studies reporting that increasing agitation speed increases aeration, which in turn increases glycerol consumption and cell growth [37]. And it was also observed that the molar conversion to (*R*)-acetoin increased with increasing agitation speed. The reason for these results was that as the agitation speed increased, a large amount of (*R*)-acetoin, a precursor of (*R,R*)-2,3-BDO, was produced (Table 3 and Table S3), but the corresponding conversion from (*R*)-acetoin to (*R,R*)-2,3-BDO was insufficient, so the accumulated (*R*)-acetoin was gradually converted to *meso*-2,3-BDO, which was assumed to have reduced the purity of (*R,R*)-2,3-BDO. In another study on the production of (*R,R*)-2,3-BDO derived from glucose using *K. oxytoca*, it was reported that *meso*-2,3-BDO was produced despite the removal of *budC* due to several other pathways to generate *meso*-2,3-BDO [10].

**Fig. 4:**
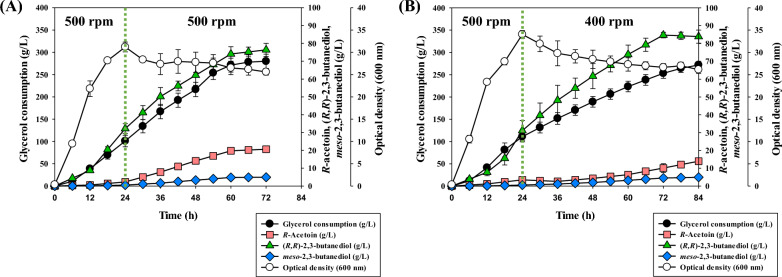
Fed-batch fermentation for glycerol-derived (*R,R*)-2,3-BDO production of *K. pneumoniae* GEM167*ΔadhEΔldhAΔbudC* according to agitation strategy. Fed-batch cultivation was in a 5-L jar fermentor (37 °C, 2.0 vvm, and pH 6.0 maintained using 28% v/v NH_4_OH) for 84 h. **A** Single agitation speed method (maintained at 500 rpm); **B** Two-stage agitation speed method (adjusted from 500 rpm of initial agitation speed to 400 rpm after 24 h)

In addition, when cultured for a long period of time at the selected agitation speed of 500 rpm for 72 h, it was observed that the purity of (*R,R*)-2,3-BDO rapidly dropped after 24 h as shown in Table 4. And Fig. 4A showed that the accumulation of acetoin gradually increased over time in the culture. After 72 h, the amount of (*R,R*)-2,3-BDO produced stagnated, and the cultivation was terminated. These results suggested that in order to maintain high purity of (*R,R*)-2,3-BDO, the amount of acetoin generated was required to be controlled. To achieve this, a two-step agitation speed control strategy was employed to mitigate the drop in purity of (*R,R*)-2,3-BDO based on the production and molar conversion ratio of acetoin according to agitation speed (Table 3 and Table S3). Given that the purity of (*R,R*)-2,3-BDO rapidly decreased after 36 h of cultivation (data was not shown), the agitation speed was lowered to 400 rpm and 300 rpm, respectively, after 24 h of cultivation.

**Table 4:** Effects of two-stage agitation strategy on 2,3-butanediol biosynthesis in *K. pneumoniae* GEM167*ΔadhEΔldhAΔbudC* after 72 h

Two-stage agitation speed (rpm)		Time (h)	Metabolites (g/L)					Purity of (*R,R*)-2,3-BDO (%)
			(*R*)-acetoin	(*S*)-acetoin	2,3-Butanediol isomers			
Initial	After 24 h				(*S,S*)-	(*R,R*)-	*Meso*-	
500	500	24	2.29 ± 0.77	–	–	32.19 ± 2.73	0.51 ± 0.07	98.45 ± 0.10
		48	14.11 ± 0.32	–	–	62.16 ± 5.68	3.29 ± 0.08	94.95 ± 0.32
		72	20.63 ± 1.27	0.93 ± 0.11	–	76.39 ± 3.65	4.94 ± 0.03	93.93 ± 0.24
	400	24	3.48 ± 0.64	–	–	31.12 ± 5.69	0.59 ± 0.06	98.15 ± 0.14
		48	4.32 ± 0.05	–	–	61.68 ± 6.99	2.03 ± 0.20	96.81 ± 0.04
		72	10.12 ± 2.48	–	–	84.73 ± 1.58	4.53 ± 0.03	94.92 ± 0.12
	300	24	2.30 ± 0.89	–	–	29.32 ± 4.11	0.46 ± 0.04	98.46 ± 0.08
		48	1.39 ± 0.37	–	–	50.18 ± 5.92	0.97 ± 0.11	98.10 ± 0.01
		72	2.58 ± 0.66	–	–	67.67 ± 3.46	1.83 ± 0.09	97.37 ± 0.01^a^

-, not detected

^a^Fed-batch cultivation was carried out at 37 ℃, 2.0 vvm, pH 6 control (using ammonia water), and agitation speed was adjusted from 500 rpm initially to either 400 rpm or 300 rpm after 24 h

As a consequence, it was proven that with adjustment of the agitation speed to two stages (500 rpm to either 400 rpm or 300 rpm after 24 h), the rate of decline in the purity of (*R,R*)-2,3-BDO was reduced when compared to the previous condition in which the agitation speed was kept at 500 rpm, as shown in Table 4. Accordingly, the duration to maintain purity of (*R,R*)-2,3-BDO at 98% was extended. Furthermore, as the agitation speed was reduced to 400 rpm or 300 rpm after 24, the amount of acetoin accumulated temporarily either dropped or stayed constant (Table 4). However, eventually, it was observed that the amount of acetoin accumulated rose and the purity of (*R,R*)-2,3-BDO reduced as the cultivation period progressed. It could be speculated to be the result of the accumulation of metabolites in the cell in the later stages of cultivation and the decrease in cell activity, resulting in the intracellular metabolism does not work properly. Nevertheless, it was indicated that using a two-stage agitation speed control strategy led to an increase in the molar conversion to (*R,R*)-2,3-BDO in Table S4 (see Additional file 1). Furthermore, the production of (*R,R*)-2,3-BDO increased from 76.39 ± 3.65 g/L to 84.73 ± 1.58 g/L when the agitation speed adjusted from 500 to 400 rpm as compared with using as single agitation speed (500 rpm) (Table 4). And the highest molar conversion to (*R,R*)-2,3-BDO obtained (0.36 ± 0.02 mol/mol of glycerol) when the agitation speed was adjusted from 500 to 300 rpm (Additional file 1: Table S4), but the production titer of (*R,R*)-2,3-BDO was 67.67 ± 3.46 g/L, which was lower than the titer when a single agitation speed was used (Table 4). Therefore, considering that the purity of (*R,R*)-2,3-BDO inevitably decreased in the later stage of cultivation even if the agitation speed was lowered to 300 rpm, the two-step agitation speed control method that showed the highest titer of (*R,R*)-2,3-BDO was chosen, which was to adjust the agitation speed from 500 to 400 rpm. It has been reported that other studies have employed a two-step agitation speed control strategy to reduce acetoin accumulation and enhance the production titer and yield of 2,3-BDO [38]. However, the highest purity of (*R,R*)-2,3-BDO still remained around 98%. It could be suggested that for the production of (*R,R*)-2,3-BDO of higher purity, overexpression of the *dhaD* gene involved in (*R,R*)-2,3-BDO biosynthesis (Fig. 3) would be beneficial.

### Enhancement of *(R,R)*-2,3-BDO purity by overexpression of *dhaD* gene associated with biosynthesis of *(R,R)*-2,3-BDO

To overcome the limited purity of (*R,R*)-2,3-BDO, we attempted to overexpress the *dhaD* gene involved in (*R,R*)-2,3-BDO biosynthesis. First, to overexpress *dhaD* gene in *K. pneumoniae* GEM167 *ΔadhEΔldhAΔbudC* strain, *dhaD*/pETM6-Tet. plasmid was introduced into *K. pneumoniae* GEM167 *ΔadhEΔldhAΔbudC* strain, and as shown in Fig. S2, *K. pneumoniae* GEM167 *ΔadhEΔldhAΔbudC*-*dhaD*/pETM6 strain was prepared. Then, it was investigated if higher purity of (*R,R*)-2,3-BDO production was possible by effectively converting (*R*)-acetoin accumulated to (*R,R*)-2,3-BDO by overexpressing *dhaD* gene with a two-step agitation speed control method (from 500 to 400 rpm after 24 h). The cultivation was carried out at 37℃, 2.0 vvm, pH 6 control (using ammonia water) for 84 h, and the agitation speed was adjusted from 500 rpm initially to 400 rpm after 24 h. In addition, the *dhaD* gene using the T7 promoter in the pETM6 plasmid was overexpressed by addition of IPTG after 6 h as an inducer.

**Fig. 5:**
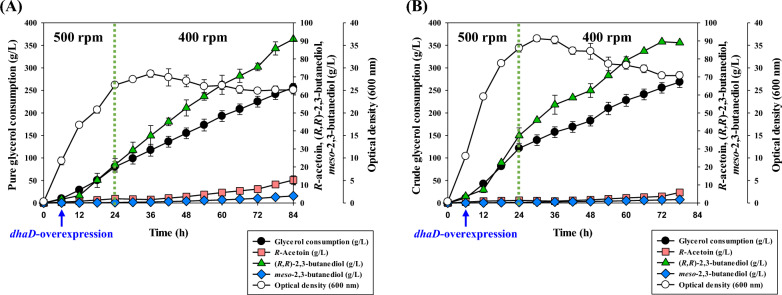
Fed-batch fermentation for (*R,R*)-2,3-BDO production from pure glycerol (or crude glycerol) in *K. pneumoniae* GEM167*ΔadhEΔldhAΔbudC*-*dhaD*/pETM6. Fed-batch cultivation was in a 5-L jar fermentor (37 °C, 2.0 vvm, and pH 6.0 maintained using 28% v/v NH_4_OH) for 84 h along with a two-step agitation speed control method (adjusted from 500 to 400 rpm after 24 h). The *dhaD* gene using the T7 promoter in the pETM6-Tet. plasmid was overexpressed by addition of IPTG after 6 h as an inducer. **A** pure glycerol; **B** crude glycerol

As a result, as shown in Table 5, overexpression of the *dhaD* gene prolonged the period during which 98% purity of (*R,R*)-2,3-BDO was maintained from 24 to 48 h compared to the control group where *dhaD* was not overexpressed (Table 4). As seen in Table S5 (see Additional file 1), when the *dhaD* gene was overexpressed, the molar conversion rate from the moles of 2,3-BDO isomers [(*R,R*)-2,3-BDO and *meso*-2,3-BDO] to (*R,R*)-2,3-BDO was 97.14% [mol of (*R,R*)-2,3-BDO/mol of (*R,R*)- + *meso*-2,3-BDO], and it was 94.44% [mol of (*R,R*)-2,3-BDO/mol of (*R,R*)- + *meso*-2,3-BDO] in the control group where *dhaD* was not overexpressed (Additional file 1: Table S4). This indicated that overexpression of *dhaD* had a positive effect on enhancing the molar conversion rate to (*R,R*)-2,3-BDO. Moreover, in the later stage of cultivation when *dhaD* was overexpressed, the maximal (*R,R*)-2,3-BDO production was 90.96 ± 0.23 g/L, which was higher than the control group without overexpression of *dhaD* (84.73 ± 1.58 g/L) (Figs. 4B and 5A).

**Table 5:** Effects of carbon source on 2,3-butanediol biosynthesis in *K. pneumoniae ΔadhEΔldhAΔbudC*-*dhaD*/pETM6 under two-stage agitation strategy

Carbon sources	Time (h)	Metabolites (g/L)					Purity of (*R,R*)-2,3-BDO (%)
		(*R*)-acetoin	(*S*)-acetoin	2,3-Butanediol isomers			
				(*S,S*)-	(*R,R*)-	*Meso*-	
Pure glycerol	24	2.36 ± 0.59	–	–	21.23 ± 3.43	0.27 ± 0.03	98.74 ± 0.06
	48	3.50 ± 0.14	–	–	52.79 ± 4.46	1.04 ± 0.14	98.07 ± 0.10
	72	7.70 ± 1.93	–	–	75.53 ± 2.10	2.53 ± 0.09	96.76 ± 0.02
	84	12.74 ± 2.34	–	–	90.96 ± 0.23	3.92 ± 0.17	95.87 ± 0.16
Crude glycerol	24	1.44 ± 0.44	–	–	37.40 ± 4.32	0.36 ± 0.05	99.05 ± 0.02
	48	1.74 ± 0.20	–	–	62.43 ± 3.81	0.84 ± 0.16	98.67 ± 0.17
	72	3.63 ± 0.73	–	–	89.47 ± 0.11	1.69 ± 0.07	98.15 ± 0.07
	78	5.84 ± 1.03	–	–	88.98 ± 0.20	1.87 ± 0.03	97.94 ± 0.03^a^

-, not detected

^a^Fed-batch cultivation was carried out at 37 ℃, 2.0 vvm, pH 6 control (using ammonia water), and the agitation speed was adjusted from 500 rpm initially to 400 rpm after 24 h. The *dhaD* gene using the T7 promoter in the pETM6 plasmid was overexpressed by addition of IPTG after 6 h as an inducer

In addition, Figs. 4B and 5A showed that the *K. pneumoniae* GEM167 *ΔadhEΔldhAΔbudC*-*dhaD*/pETM6 had slower initial cell growth compared to the *K. pneumoniae* GEM167 *ΔadhEΔldhAΔbudC* strain. *K. pneumoniae* GEM167 *ΔadhEΔldhAΔbudC* strain reached the maximum cell growth in 24 h, while the *K. pneumoniae* GEM167 *ΔadhEΔldhAΔbudC*-*dhaD*/pETM6 reached maximum cell growth in 36 h. And the maximum cell growth was higher in the *K. pneumoniae* GEM167 *ΔadhEΔldhAΔbudC* strain (O.D = 34.05 ± 0.85) than in the *K. pneumoniae* GEM167 *ΔadhEΔldhAΔbudC*-*dhaD*/pETM6 (O.D = 28.7 ± 0.77) (Figs. 4B and 5A). It was suggested that this was due to the burden imposed on the cells by the introduction and expression of *dhaD*/pETM6 plasmid and the addition of antibiotic addition delayed cell growth.

Unfortunately, although the overexpression of *dhaD* boosted the production of (*R,R*)-2,3-BDO while maintaining 98% purity of (*R,R*)-2,3-BDO for a longer period of culture time than before, it still did not prevent purity of (*R,R*)-2,3-BDO from dropping below 98% in the late stages of cultivation (Table 5). Even when *dhaD* was not overexpressed, the amounts of *meso*-2,3-BDO were small, but as it was generated gradually, the purity of (*R,R*)-2,3-BDO decreased gradually, suggesting that these results might be related to the limitation of *dhaD* gene in selectively converting from (*R*)-acetoin to (*R,R*)-2,3-BDO. Because it has been reported that the activity of converting (*S*)-acetoin to *meso*-2,3-BDO is greater than that of converting of (*R*)-acetoin to (*R,R*)-2,3-BDO in whole-cell biocatalysis using (*R*)/(*S*)-acetoin as substrate employing *K. pneumoniae ΔbudCΔgldA* [13]. Thus, although the overexpression of *dhaD* was able to increase the production of (*R,R*)-2,3-BDO, it was speculated that the *dhaD* gene might be limited in maintaining a higher purity of (*R,R*)-2,3-BDO due to the accumulation of *meso*-2,3-BDO, which is gradually generated in small amounts.

As shown in Table 6, previous studies have indicated that the selective production of (*R,R*)-2,3-BDO using a *Klebsiella* sp. strain resulted in a maximum (*R,R*)-2,3-BDO purity of up to 98% or less, even with the expression of exogenous genes. From this, it can be inferred that exogenous genes overexpressed in previous studies might have limitations in their ability to produce (*R,R*)-2,3-BDO with higher purity. However, many studies have been conducted on the properties of 2,3-BDO dehydrogenases derived from various species [12], as well as the 2,3-BDO dehydrogenase used in previous studies. Therefore, it could be suggested that higher purity of (*R,R*)-2,3-BDO could be achieved if enzymes that specifically convert (*R*)-acetoin to (*R,R*)-2,3-BDO are utilized.

**Table 6:** Comparison with previous studies on (*R,R*)-2,3-butanediol production using various carbon sources in *Klebsiella* sp. strains

Microorganism	Isomer	Carbon source	Fermentation type	Concentration (g/L)	(*R,R*)-2,3-BDO productivity (g/L/h)	(*R,R*)-2,3-BDO yield (g/g)	References
*Klebsiella oxytoca ΔldhAΔpflBΔbudC*::PBDH (pBBR-PBDH)	(*R,R*)- (92%)	Glucose	Fed-batch	106.7 (*R,R-*), 9.3 (*Meso-*)	3.1	0.40	[10]
*Klebsiella pneumoniae ΔwabGΔbudCΔldhA*::*gldA*::*dhaD*	(*R,R*)- (98%)	Glucose	Fed-batch	61 (*R,R-*), 1.4 (*Meso-*)	0.51	0.36	[14]
*K. pneumoniae* GEM167*ΔadhEΔldhAΔbudC*::*dhaD*	(*R,R*)- (96%)	Pure glycerol	Fed-batch	90.96 (*R,R-*), 3.92 (*Meso-*)	1.08	0.35	This study
*K. pneumoniae* GEM167*ΔadhEΔldhAΔbudC*::*dhaD*	(*R,R*)- (98%)	Crude glycerol	Fed-batch	89.47 (*R,R-*), 1.69 (*Meso-*)	1.24	0.35	This study

### Substitutability to crude glycerol

As mentioned above, the significance of biorefinery research employing industrial waste or non-food resources as biomass is growing, and it is essential to establish a virtuous cycle system between the two processes by securing such biomass and using this biomass for biorefinery [1]. Therefore, in this study, it was investigated whether crude glycerol, an industrial waste from the biodiesel production process, could be replaced with pure glycerol to produce cost-effectively (*R,R*)-2,3-BDO. To this end, pure glycerol was replaced with crude glycerol as a sole carbon source and, the *K. pneumoniae* GEM167 *ΔadhEΔldhAΔbudC*-*dhaD*/pETM6 strain was cultured as mentioned above.

As a result, as indicated in Table 5, the purity of (*R,R*)-2,3-BDO was maintained at 98% for 72 h, and the maximum (*R,R*)-2,3-BDO production using crude glycerol was 89.47 ± 0.11 g/L at 72 h of cultivation. And the maximum (*R,R*)-2,3-BDO production using pure glycerol (90.96 ± 0.23 g/L at 84 h) was similar to the maximum production using crude glycerol, however, replacement to crude glycerol reduced the time to attain its maximum production by 12 h. As a result, the productivity of (*R,R*)-2,3-BDO increased from 1.08 g/L/h (using pure glycerol) to 1.24 g/L/h (using crude glycerol). The yield for each maximal (*R,R*)-2,3-BDO production was identical at 0.35 g (*R,R*)-2,3-BDO/g of consumed glycerol. The result in Fig. 5 showed that maximal cell growth using crude glycerol was OD = 36.5 ± 0.66, whereas maximum cell growth using pure glycerol was OD = 28.7 ± 0.85. Furthermore, cell growth in the early phases of cultivation was considerably accelerated when crude glycerol was used compared to pure glycerol (Fig. 5). These results have been suggested to be caused by the reason that crude glycerol includes certain components that encourage cell growth [39]. And previous study has demonstrated that using crude glycerol improved 2,3-BDO production and productivity [36]. Table S5 (see Additional file 1) demonstrated that the molar conversion rate to (*R,R*)-2,3-BDO from crude glycerol was higher than that from pure glycerol at 72 h of cultivation. It was observed that the molar conversion rate to (*R*)-acetoin decreased from 0.04 ± 0.01 mol/mol of consumed pure glycerol to 0.01 ± 0.01 mol/mol of consumed crude glycerol, indicating that the conversion from (*R*)-acetoin to (*R,R*)-2,3-BDO was efficiently carried out when crude glycerol was used. Although not shown by data, the molar conversion rate to (*R,R*)-2,3-BDO at 84 h of cultivation, which exhibited the maximum (*R,R*)-2,3-BDO, increased to 0.36 ± 0.00 mol/mol of consumed pure glycerol when pure glycerol. However, not only this, the molar conversion rate to both *meso*-2,3-BDO (from 0.01 ± 0.00 to 0.02 ± 0.01 mol/mol of consumed pure glycerol) and (*R*)-acetoin (from 0.04 to 0.05 mol/mol of consumed pure glycerol) increased. Given the molar conversion rate to metabolites in Table S5 (see Additional file 1), further study would be expected to have a positive effect on (*R,R*)-2,3-BDO production by restricting the pathway to metabolites such as succinate among competitive metabolites, hence preventing carbon flux dispersion.

As shown in Table 6, it was shown that only glucose has been used to produce (*R,R*)-2,3-BDO in the metabolically engineered *Klebsiella* sp. strains. In previous studies, *Bacillus* sp. [40, 41] and *Panebacillus* sp. strains [42, 43] that originally produced only (*R,R*)-2,3-BDO, have been used to produce (*R,R*)-2,3-BDO. However, since it is difficult for these strains to use glycerol as a sole carbon source, there were few studies on the production of (*R,R*)-2,3-BDO from glycerol. Meanwhile, there are also only a few studies on (*R,R*)-2,3-BDO using *Klebsiella* sp. strains, which can be metabolized with glycerol alone in Table 6. This could be because of the comparatively low purity of (*R,R*)-2,3-BDO and the formation of 1,3-PDO, a major byproduct of the glycerol metabolism in *Klebsiella* sp. strain. Nevertheless, in this study, mass production of the highest purity of (*R,R*)-2,3-BDO was achieved for the first time using glycerol as the sole carbon source.

In addition, it was meaningful to confirm the feasibility of substituting pure glycerol with crude glycerol. This is due to the reason that crude glycerol is a more readily available raw material and less expensive. And since large-scale industrial facilities and refining costs are required to purify crude glycerol into pure glycerol, replacing commercial pure glycerol with crude glycerol, which is easily obtained from the biodiesel production process, could lower costs by more than 20-fold [44]. Because the cost of feedstock for carbon source contributes to bulk chemical production by up to 50% [45], cost competitiveness obtained from the use of crude glycerol could be a driving force behind the commercialization of glycerol-derived (*R,R*)-2,3-BDO production. Previous studies have also suggested that cultivation using a low-nutritional medium was required to be more economically feasible, so cultivation using industrially diverse biomass other than glucose was required [14]. Therefore, this study is expected to be valuable in that it suggested the direction for further study for glycerol-derived (*R,R*)-2,3-BDO production as well as the achievement of high-concentration production of high purity (*R,R*)-2,3-BDO using glycerol.

## Conclusions

The major isomer form of 2,3-BDO produced in *K. pneumoniae* GEM167 with an enhanced oxidative pathway was *meso*-2,3-BDO. In this study, the *budC* gene was deleted to selectively produce (*R,R*)-2,3-BDO with high utilization value. This resulted in the major isomer form of 2,3-BDO shifts from *meso*-2,3-BDO to (*R,R*)-2,3-BDO. To improve the purity of (*R,R*)-2,3-BDO, the endogenous *dhaD* gene involved in (*R,R*)-2,3-BDO biosynthesis was overexpressed in *K. pneumoniae* along with a two-step adjustment of agitation speed. And the crude glycerol was successfully replaced with pure glycerol with no negative effects, allowing not only the cost-effective production of (*R,R*)-2,3-BDO but also increasing the production of (*R,R*)-2,3-BDO with high purity due to cell growth boosted by the replacement to crude glycerol. This is the first study to selectively produce (*R,R*)-2,3-BDO without production of 1,3-PDO despite glycerol-fermentation by *K. pneumoniae* strain due to the unique characteristics of *K. pneumoniae* GEM167 mutant. Moreover, (*R,R*)-2,3-BDO of the highest purity was obtained in the highest titer using glycerol from *K. pneumoniae*.

### Supplementary Information

Supplementary material 1.

## Data Availability

All data generated or analyzed during this study are included in this published article [and its Addiitonal files].

## References

[1] Almeida JR, Fávaro LC, Quirino BF. Biodiesel biorefinery: opportunities and challenges for microbial production of fuels and chemicals from glycerol waste. Biotechnol Biofuels. 2012;5:48. 22809320 10.1186/1754-6834-5-48 PMC3467170

[2] Bai Y, Feng H, Liu N, Zhao X. Biomass-derived 2, 3-butanediol and its application in biofuels production. Energies. 2023;16:5802. 10.3390/en16155802

[3] Costa-Gutierrez SB, Saez JM, Aparicio JD, Raimondo EE, Benimeli CS, Polti MA. Glycerol as a substrate for actinobacteria of biotechnological interest: advantages and perspectives in circular economy systems. Chemosphere. 2021;279:130505. 33865166 10.1016/j.chemosphere.2021.130505

[4] Jiang Y, Ye X, Zheng T, Dong W, Xin F, Ma J, Jiang M. Microbial production of L-malate from renewable non-food feedstocks. Chin J Chem Eng. 2021;30:105–11. 10.1016/j.cjche.2020.10.017

[5] Talan A, Pokhrel S, Tyagi R, Drogui P. Biorefinery strategies for microbial bioplastics production: sustainable pathway towards Circular Bioeconomy. Bioresour Technol Rep. 2022;17:100875. 10.1016/j.biteb.2021.100875

[6] Yang F, Hanna MA, Sun R. Value-added uses for crude glycerol–a byproduct of biodiesel production. Biotechnol Biofuels. 2012;5:1–10. 22413907 10.1186/1754-6834-5-13 PMC3313861

[7] Da Silva GP, Mack M, Contiero J. Glycerol: a promising and abundant carbon source for industrial microbiology. Biotechnol Adv. 2009;27:30–9. 18775486 10.1016/j.biotechadv.2008.07.006

[8] Li C, Lesnik KL, Liu H. Microbial conversion of waste glycerol from biodiesel production into value-added products. Energies. 2013;6:4739–68. 10.3390/en6094739

[9] Park JM, Hong W-K, Lee S-M, Heo S-Y, Jung YR, Kang IY, Oh B-R, Seo J-W, Kim CH. Identification and characterization of a short-chain acyl dehydrogenase from Klebsiella pneumoniae and its application for high-level production of L-2, 3-butanediol. J Ind Microbiol Biotechnol. 2014;41:1425–33. 25037723 10.1007/s10295-014-1483-7

[10] Park JM, Rathnasingh C, Song H. Enhanced production of (R, R)-2, 3-butanediol by metabolically engineered Klebsiella oxytoca. J Ind Microbiol Biotechnol. 2015;42:1419–25. 26275527 10.1007/s10295-015-1648-z

[11] Wang Y, Tao F, Li C, Li L, Xu P. Genome sequence of Klebsiella pneumoniae strain ATCC 25955, an oxygen-insensitive producer of 1, 3-propanediol. Genome Announc. 2013. 10.1128/genomea. 23929480 10.1128/genomea PMC3738896

[12] Yang Z, Zhang Z. Recent advances on production of 2, 3-butanediol using engineered microbes. Biotechnol Adv. 2019;37:569–78. 29608949 10.1016/j.biotechadv.2018.03.019

[13] Wang Y, Tao F, Xu P. Glycerol dehydrogenase plays a dual role in glycerol metabolism and 2, 3-butanediol formation in Klebsiella pneumoniae. J Biol Chem. 2014;289:6080–90. 24429283 10.1074/jbc.M113.525535 PMC3937674

[14] Lee S, Kim B, Yang J, Jeong D, Park S, Lee J. A non-pathogenic and optically high concentrated (R, R)-2, 3-butanediol biosynthesizing Klebsiella strain. J Biotechnol. 2015;209:7–13. 26074000 10.1016/j.jbiotec.2015.06.385

[15] Celińska E, Grajek W. Biotechnological production of 2, 3-butanediol—current state and prospects. Biotechnol Adv. 2009;27:715–25. 19442714 10.1016/j.biotechadv.2009.05.002

[16] Sadhu KM, Matteson DS, Hurst GD, Kurosky JM. (R, R)-2, 3-Butanediol as chiral directing group in the synthesis of (S)-. alpha.-chloro boronic esters. Organometallics. 1984;3:804–6. 10.1021/om00083a028

[17] Yu B, Sun J, Bommareddy RR, Song L, Zeng A-P. Novel (2 R, 3 R)-2, 3-butanediol dehydrogenase from potential industrial strain Paenibacillus polymyxa ATCC 12321. Appl Environ Microbiol. 2011;77:4230–3. 21531839 10.1128/AEM.02998-10 PMC3131630

[18] Garg S, Jain A. Fermentative production of 2, 3-butanediol: a review. Biores Technol. 1995;51:103–9. 10.1016/0960-8524(94)00136-O

[19] Zeng A-P, Sabra W. Microbial production of diols as platform chemicals: recent progresses. Curr Opin Biotechnol. 2011;22:749–57. 21646010 10.1016/j.copbio.2011.05.005

[20] Gao J, Yang HH, Feng XH, Li S, Xu H. A 2, 3-butanediol dehydrogenase from Paenibacillus polymyxa ZJ-9 for mainly producing R, R-2, 3-butanediol: purification, characterization and cloning. J Basic Microbiol. 2013;53:733–41. 22961752 10.1002/jobm.201200152

[21] Ryu C-M, Farag MA, Hu C-H, Reddy MS, Kloepper JW, Paré PW. Bacterial volatiles induce systemic resistance in Arabidopsis. Plant Physiol. 2004;134:1017–26. 14976231 10.1104/pp.103.026583 PMC389924

[22] Kumar V, Sankaranarayanan M, Jae K-E, Durgapal M, Ashok S, Ko Y, Sarkar R, Park S. Co-production of 3-hydroxypropionic acid and 1, 3-propanediol from glycerol using resting cells of recombinant Klebsiella pneumoniae J2B strain overexpressing aldehyde dehydrogenase. Appl Microbiol Biotechnol. 2012;96:373–83. 22684326 10.1007/s00253-012-4187-9

[23] Maina S, Prabhu AA, Vivek N, Vlysidis A, Koutinas A, Kumar V. Prospects on bio-based 2, 3-butanediol and acetoin production: recent progress and advances. Biotechnol Adv. 2022;54:107783. 34098005 10.1016/j.biotechadv.2021.107783

[24] Xiu Z-L, Zeng A-P. Present state and perspective of downstream processing of biologically produced 1, 3-propanediol and 2, 3-butanediol. Appl Microbiol Biotechnol. 2008;78:917–26. 18320188 10.1007/s00253-008-1387-4

[25] Jo M-H, Ju J-H, Heo S-Y, Cho J, Jeong KJ, Kim M-S, Kim C-H, Oh B-R. Production of 1, 2-propanediol from glycerol in Klebsiella pneumoniae GEM167 with flux enhancement of the oxidative pathway. Biotechnol Biofuels Bioproducts. 2023;16:18. 10.1186/s13068-023-02269-4 PMC9903448 36747250

[26] Oh B-R, Seo J-W, Heo S-Y, Hong W-K, Luo LH, Joe M-H, Park D-H, Kim CH. Efficient production of ethanol from crude glycerol by a Klebsiella pneumoniae mutant strain. Bioresour Technol. 2011;102:3918–39222. 21186120 10.1016/j.biortech.2010.12.007

[27] Datsenko KA, Wanner BL. One-step inactivation of chromosomal genes in Escherichia coli K-12 using PCR products. Proc Natl Acad Sci. 2000;97:6640–5. 10829079 10.1073/pnas.120163297 PMC18686

[28] Cherepanov PP, Wackernagel W. Gene disruption in Escherichia coli: TcR and KmR cassettes with the option of Flp-catalyzed excision of the antibiotic-resistance determinant. Gene. 1995;158:9–14. 7789817 10.1016/0378-1119(95)00193-A

[29] Oh B-R, Lee S-M, Heo S-Y, Seo J-W, Kim CH. Efficient production of 1, 3-propanediol from crude glycerol by repeated fed-batch fermentation strategy of a lactate and 2, 3-butanediol deficient mutant of Klebsiella pneumoniae. Microb Cell Fact. 2018;17:1–9. 29907119 10.1186/s12934-018-0921-z PMC6003044

[30] Seo M-Y, Seo J-W, Heo S-Y, Baek J-O, Rairakhwada D, Oh B-R, Seo P-S, Choi MH, Kim CH. Elimination of by-product formation during production of 1, 3-propanediol in Klebsiella pneumoniae by inactivation of glycerol oxidative pathway. Appl Microbiol Biotechnol. 2009;84:527–34. 19352645 10.1007/s00253-009-1980-1

[31] Fournet-Fayard S, Joly B, Forestier C. Transformation of wild type Klebsiella pneumoniae with plasmid DNA by electroporation. J Microbiol Methods. 1995;24:49–54. 10.1016/0167-7012(95)00053-4

[32] Livak KJ, Schmittgen TD. Analysis of relative gene expression data using real-time quantitative PCR and the 2− ΔΔCT method. Methods. 2001;25:402–8. 11846609 10.1006/meth.2001.1262

[33] Ju J-H, Heo S-Y, Choi S-W, Kim Y-M, Kim M-S, Kim C-H, Oh B-R. Effective bioconversion of 1, 3-propanediol from biodiesel-derived crude glycerol using organic acid resistance–enhanced Lactobacillus reuteri JH83. Biores Technol. 2021;337:125361. 10.1016/j.biortech.2021.125361 34320778

[34] Priya A, Dureja P, Talukdar P, Rathi R, Lal B, Sarma PM. Microbial production of 2, 3-butanediol through a two-stage pH and agitation strategy in 150 l bioreactor. Biochem Eng J. 2016;105:159–67. 10.1016/j.bej.2015.09.016

[35] Maina S, Mallouchos A, Nychas GJE, Freire DM, de Castro AM, Papanikolaou S, Kookos IK, Koutinas A. Bioprocess development for (2R, 3R)-butanediol and acetoin production using very high polarity cane sugar and sugarcane molasses by a Bacillus amyloliquefaciens strain. J Chem Technol Biotechnol. 2019;94:2167–77. 10.1002/jctb.5997

[36] Jo M-H, Ju J-H, Heo S-Y, Son C-B, Jeong KJ, Oh B-R. Production of high levels of 2, 3-butanediol from renewable crude glycerol by Klebsiella pneumoniae GEM167 using metabolic engineering and control of oxygen transfer efficiency. Renew Energy. 2024;230:120793. 10.1016/j.renene.2024.120793

[37] Jo M-H, Ju J-H, Heo S-Y, Cho J, Jeong KJ, Kim M-S, Kim C-H, Oh B-R. Production of 1, 2-propanediol from glycerol in Klebsiella pneumoniae GEM167 with flux enhancement of the oxidative pathway. Biotechnol Biofuels Bioproducts. 2023;16:1–12. 10.1186/s13068-023-02269-4 PMC9903448 36747250

[38] Ji X-J, Huang H, Du J, Zhu J-G, Ren L-J, Hu N, Li S. Enhanced 2, 3-butanediol production by Klebsiella oxytoca using a two-stage agitation speed control strategy. Biores Technol. 2009;100:3410–4. 10.1016/j.biortech.2009.02.031 19297148

[39] Cho S, Kim T, Woo HM, Kim Y, Lee J, Um Y. High production of 2, 3-butanediol from biodiesel-derived crude glycerol by metabolically engineered Klebsiella oxytoca M1. Biotechnol Biofuels. 2015;8:1–12. 26379778 10.1186/s13068-015-0336-6 PMC4570460

[40] Ge Y, Li K, Li L, Gao C, Zhang L, Ma C, Xu P. Contracted but effective: production of enantiopure 2, 3-butanediol by thermophilic and GRAS Bacillus licheniformis. Green Chem. 2016;18:4693–703. 10.1039/C6GC01023G

[41] Maina S, Schneider R, Alexandri M, Papapostolou H, Nychas G-J, Koutinas A, Venus J. Volumetric oxygen transfer coefficient as fermentation control parameter to manipulate the production of either acetoin or D-2, 3-butanediol using bakery waste. Biores Technol. 2021;335:125155. 10.1016/j.biortech.2021.125155 34015563

[42] Häßler T, Schieder D, Pfaller R, Faulstich M, Sieber V. Enhanced fed-batch fermentation of 2, 3-butanediol by Paenibacillus polymyxa DSM 365. Biores Technol. 2012;124:237–44. 10.1016/j.biortech.2012.08.047 22989651

[43] Ma K, He M, You H, Pan L, Wang Z, Wang Y, Hu G, Cui Y, Maeda T. Improvement of (R, R)-2, 3-butanediol production from corn stover hydrolysate by cell recycling continuous fermentation. Chem Eng J. 2018;332:361–9. 10.1016/j.cej.2017.09.097

[44] Chakravarty S, Mallick N. Carbon dioxide mitigation and biodiesel production by a marine microalga under mixotrophic mode by using transesterification by-product crude glycerol: a synergy of biofuels and waste valorization. Environ Technol Innov. 2022;27:102441. 10.1016/j.eti.2022.102441

[45] Zhou S, Zhang Y, Wei Z, Park S. Recent advances in metabolic engineering of microorganisms for the production of monomeric C3 and C4 chemical compounds. Bioresour Technol. 2023;377:128973. 36972803 10.1016/j.biortech.2023.128973

