# Supplementary material for: High production of enantiopure (R,R)-2,3-butanediol from crude glycerol by Klebsiella pneumoniae with an engineered oxidative pathway and a two-stage agitation strategy
Source: Microb Cell Fact. 2024 Jul 23;23:205. doi: 10.1186/s12934-024-02480-4 (PMC11267846; doi:10.1186/s12934-024-02480-4)
Supplement: Supplementary file 1 — Supplementary material 1. [file 12934_2024_2480_MOESM1_ESM.docx]

**Supplementary Materials**

**High production of enantiopure (*R,R*)-2,3-butanediol from crude glycerol by *Klebsiella pneumoniae* with an engineered oxidative pathway and a two-stage agitation strategy**

Min-Ho Jo ^a, b^, Jung-Hyun Ju ^a^, Sun-Yeon Heo ^a^, Chang-Beom Son ^a^, Ki Jun Jeong ^b^ and Baek-Rock Oh ^a, *^

^a^ Microbial Biotechnology Research Center, Jeonbuk Branch Institute, Korea Research Institute of Bioscience and Biotechnology (KRIBB), Jeongeup, Jeonbuk 56212, Republic of Korea.

^b^ Department of Chemical and Biomolecular Engineering and Institute for the BioCentury, KAIST, Daejeon 34141, Republic of Korea

*** Correspondence:**

Baek-Rock Oh, Microbial Biotechnology Research Center, Jeonbuk Branch Institute, Korea Research Institute of Bioscience and Biotechnology (KRIBB), Jeongeup, Jeonbuk 56212, Republic of Korea. Tel: +82 63 570 5117; Fax: +82 63 570 5160; E-mail: baekrock.oh@kribb.re.kr (B. R. Oh)

**Supplementary Figures**

**
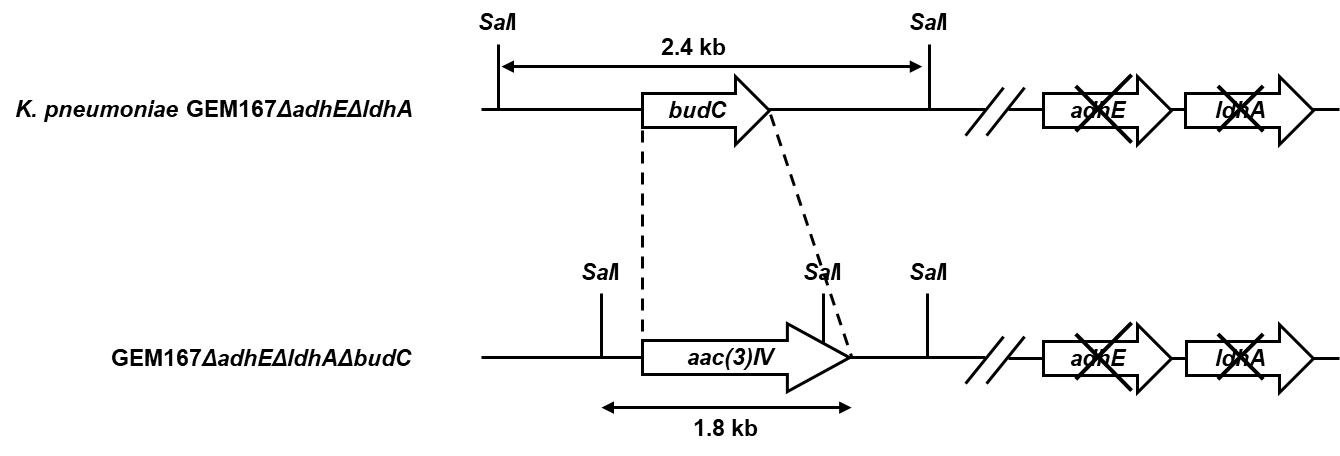
**

**Fig. S1.** Construction of the *budC*-deficient mutant of *K. pneumoniae* GEM167*ΔadhEΔldhA* by substitution of *budC* with an apramycin-resistance gene [*aac(3)IV*] via homologous recombination.


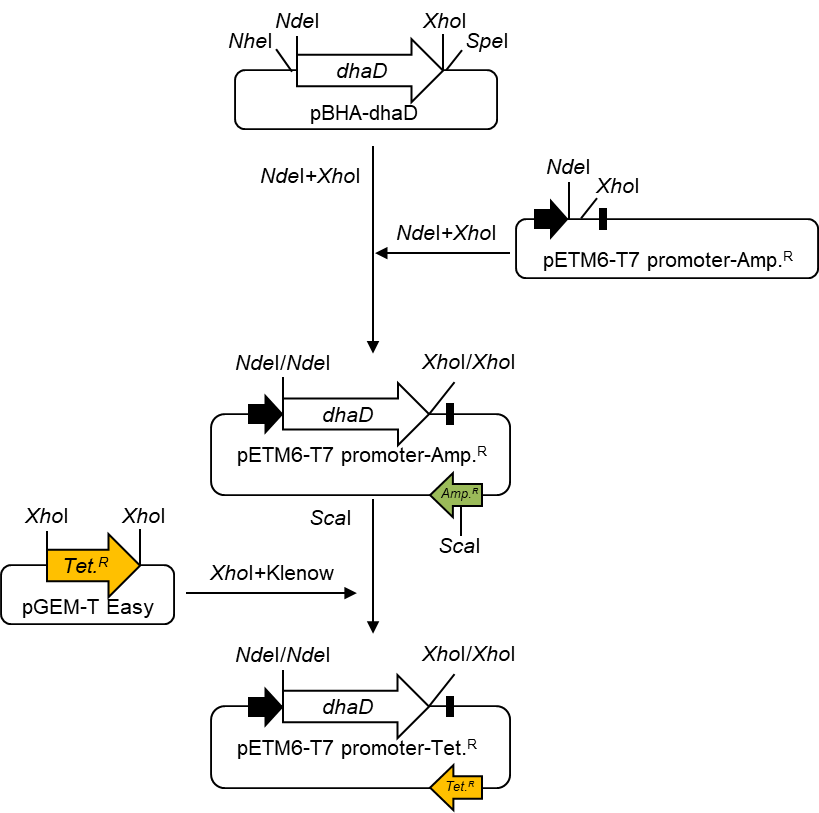


**Fig. S2.** Schematic representation of plasmid construction for *dhaD* gene expression.


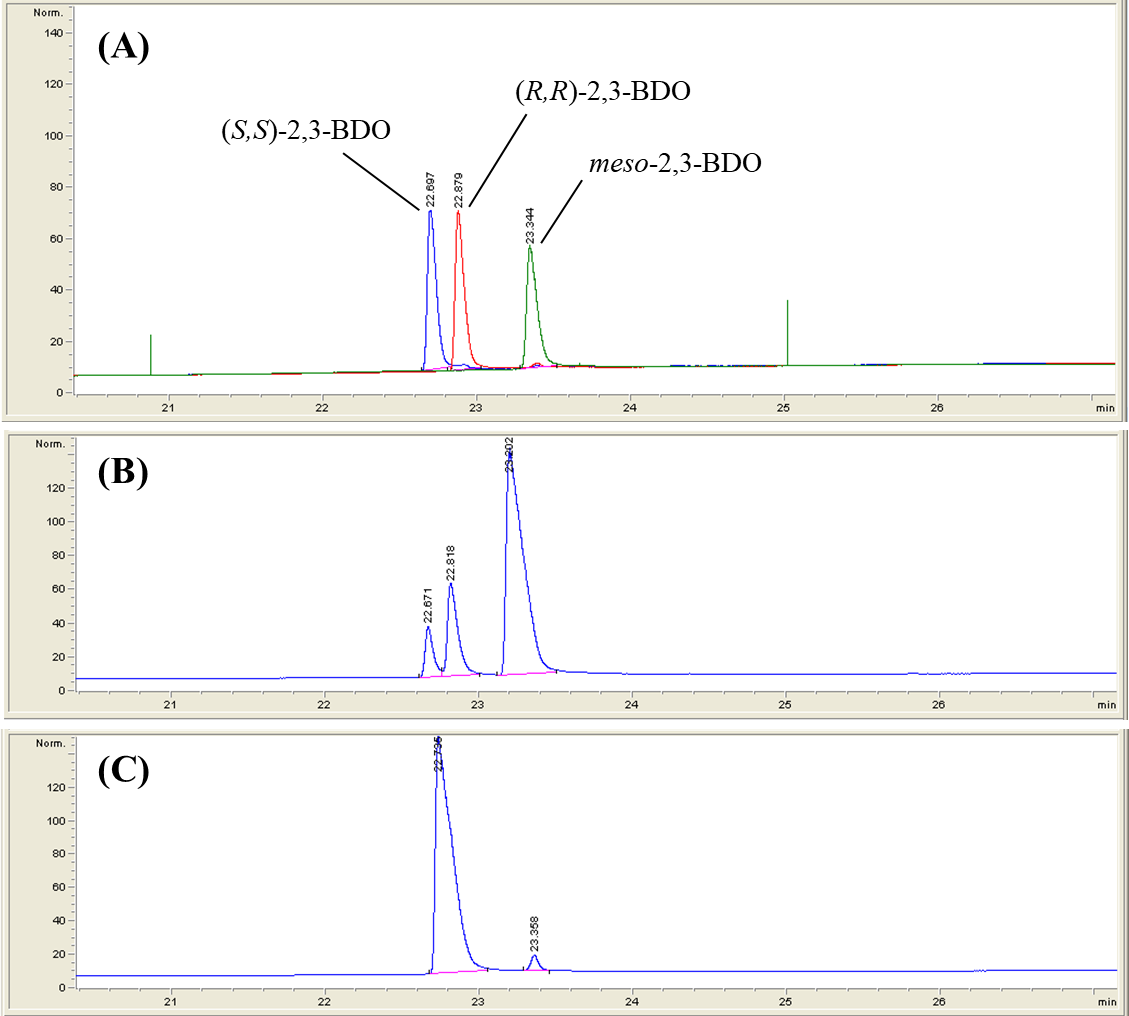


**Fig. S3.** Gas chromatography analysis results. (A) standard materials; (B) fermentation samples from *K. pneumoniae* GEM167*ΔadhEΔldhA*; (C) fermentation samples from *K. pneumoniae* GEM167*ΔadhEΔldhAΔbudC.*

**Table S1.** Oligonucleotides used in this study

| Gene | Region | Primer | Sequence (5’-3’) |
| --- | --- | --- | --- |
| *budC* | Upstream | P1 | ATCACAATAAGGAAAGGAAA |
|  |  | P2 | CGGTCATATAATCAGAATCCG*GTTAAC*CCTTTAACGTTGATGTTG |
|  |  | P5 | CCGGATGTCGAGCTGGTG |
|  | Downstream | P3 | CAACATCAACGTTAAAGG*GTTAAC*CGGATTCTGATTATATGACCG |
|  |  | P4 | ATTTGGTTCCTCAATTTTATAG |
|  |  | P6 | TATCCAATGGAATTTATGAGGG |

Italicized letters indicate restriction enzyme sites (GTTAAC, *Hpa*I)

**Table S2.** Oligonucleotides primers used in real-time RT-PCR experiment in this study

| Primer | Sequence (5’-3’) |
| --- | --- |
| *dhaD-F* | CCGGATATGGTGGTGATGGA |
| *dhaD-R* | GCAGCGTATCATAGCACAGG |
| *gldA-F* | GCGCAGCTGGATATCAAAGG |
| *gldA-R* | CGTAGACCTGGTCGGAATCA |
| *budB-F* | GCACCTGATCTGGGTCGATA |
| *budB-R* | CTTTCCACGGCAAACCCTTT |
| *budA-F* | GACCTTCGGCGAAATTCACA |
| *budA-R* | GCAGATTAGCCTGCAGGAAC |
| *budC-F* | AATGTGGGCCGAAATTGACC |
| *budC-R* | CTGGCAAGATAGGAGACGCA |
| *budR-F* | GGTATTTCACAGCCTCCGTT |
| *budR-R* | AGAAAGACTCTCCCGCTTCC |
| *rpoD-F* | TCCGAACGCCGAAGAAGATA |
| *rpoD-R* | TTGTCGTCATCGCTGTTGTC |

*dhaD*: glycerol dehydrogenase from *Klebsiella pneumoniae* MGH 78578

*gldA*: glycerol dehydrogenase from *Klebsiella pneumoniae* MGH 78578

*budB*: acetolactate synthase from *Klebsiella pneumoniae* MGH 78578

*budA*: alpha-acetolactate decarboxylase from *Klebsiella pneumoniae* MGH 78578

*budC*: acetoin reductase from *Klebsiella pneumoniae* MGH 78578

*budR*: transcriptional activator-like protein from *Klebsiella pneumoniae* MGH 78578

*rpoD*: RNA polymerase sigma factor from *Klebsiella pneumoniae* MGH 78578

**Table S3.** Molar conversion of metabolites in *K. pneumoniae* GEM167*ΔadhEΔldhAΔbudC* according to agitation speed after 24 h

| Agitation speed  (rpm) | Molar conversion (mol of metabolites/mol of glycerol) | | | | | | | | | O.D_600_ |
| --- | --- | --- | --- | --- | --- | --- | --- | --- | --- | --- |
|  | Succinate | Lactic acid | Acetic acid | (*R*)-  acetoin | (*S*)-acetoin | (*S,S*)-  2,3-BDO | (*R,R*)-  2,3-BDO | *Meso*-  2,3-BDO | Ethanol |  |
| 400 | 0.02 ± 0.00 | 0.00 ± 0.00 | 0.12 ± 0.01 | 0.02 ± 0.01 | - | - | 0.32 ± 0.03 | 0.01 ± 0.01 | 0.01 ± 0.00 | 23.00 ± 2.33 |
| 500 | 0.03 ± 0.00 | 0.00 ± 0.00 | 0.06 ± 0.01 | 0.03 ± 0.01 | - | - | 0.33 ± 0.02 | 0.01 ± 0.00 | 0.00 ± 0.00 | 31.20 ± 1.10 |
| 600 | 0.03 ± 0.00 | 0.00 ± 0.00 | 0.05 ± 0.01 | 0.05 ± 0.01 | - | - | 0.28 ± 0.03 | 0.01 ± 0.00 | 0.00 ± 0.00 | 40.80 ± 1.71 |
| 700 | 0.00 ± 0.00 | 0.00 ± 0.00 | 0.05 ± 0.01 | 0.07 ± 0.01 | - | - | 0.25 ± 0.01 | 0.01 ± 0.00 | 0.00 ± 0.00 | 44.10 ± 1.21^[[1]](#footnote-1)^ |

(-, not detected)

**Table S4.** Molar conversion of metabolites in *K. pneumoniae* GEM167*ΔadhEΔldhAΔbudC* according to two-stage agitation strategy

| Agitation speed  (rpm) | | Molar conversion (mol of metabolites/mol of glycerol) at 72 h | | | | | | | | | O.D_600_ |
| --- | --- | --- | --- | --- | --- | --- | --- | --- | --- | --- | --- |
|  |  | Succinate | Lactic acid | Acetic acid | (*R*)-  acetoin | (*S*)-acetoin | (*S,S*)-  2,3-BDO | (*R,R*)-  2,3-BDO | *Meso*-  2,3-BDO | Ethanol |  |
|  | 500 | 0.00 ± 0.00 | 0.00 ± 0.00 | 0.08 ± 0.01 | 0.08 ± 0.01 | 0.00 ± 0.00 | - | 0.28 ± 0.01 | 0.02 ± 0.00 | 0.00 ± 0.00 | 25.60 ± 0.71 |
| 500 | 400 | 0.02 ± 0.02 | 0.00 ± 0.00 | 0.06 ± 0.01 | 0.04 ± 0.01 | - | - | 0.34 ± 0.01 | 0.02 ± 0.00 | 0.00 ± 0.00 | 26.65 ± 0.85 |
|  | 300 | 0.04 ± 0.01 | 0.00 ± 0.00 | 0.06 ± 0.01 | 0.01 ± 0.01 | - | - | 0.36 ± 0.02 | 0.01 ± 0.00 | 0.00 ± 0.00 | 24.80 ± 0.63^[[2]](#footnote-2)^ |

(-, not detected)

**Table S5.** Molar conversion of metabolites from glycerol in *K. pneumoniae* GEM167*ΔadhEΔldhAΔbudC*-*dhaD*/pETM6 under two-stage agitation strategy

| Carbon sources | Molar conversion (mol of metabolites/mol of glycerol) at 72 h | | | | | | | | | | | | | | | O.D_600_ |
| --- | --- | --- | --- | --- | --- | --- | --- | --- | --- | --- | --- | --- | --- | --- | --- | --- |
|  | Succinate | Lactic acid | Acetic acid | (*R*)-acetoin | | (*S*)-acetoin | | (*S,S*)-  2,3-BDO | | (*R,R*)-  2,3-BDO | | *Meso*-  2,3-BDO | | Ethanol | |  |
| Pure glycerol | 0.00 ± 0.00 | 0.00 ± 0.00 | 0.04 ± 0.01 | 0.04 ± 0.01 | - | | - | | 0.34 ± 0.01 | | 0.01 ± 0.00 | | 0.00 ± 0.00 | | 24.90 ± 0.51 | |
| Crude glycerol | 0.03 ± 0.01 | 0.00 ± 0.00 | 0.05 ± 0.01 | 0.01 ± 0.01 | - | | - | | 0.36 ± 0.00 | | 0.01 ± 0.00 | | 0.00 ± 0.00 | | 28.30 ± 0.66^[[3]](#footnote-3)^ | |

(-, not detected)

1. Fed-batch cultivation was carried out in a 5-L jar fermentor (37°C, 700 rpm, 2.0 vvm, and pH 6.0 maintained using ammonium water) according to the agitation speed (400, 500, 600, and 700 rpm). [↑](#footnote-ref-1)
2. Fed-batch cultivation was carried out at 37℃, 2.0 vvm, pH 6 control (using ammonia water), and agitation speed was adjusted from 500 rpm initially to either 400 rpm or 300 rpm after 24 h. [↑](#footnote-ref-2)
3. Fed-batch cultivation was carried out at 37℃, 2.0 vvm, pH 6 control (using ammonia water), and the agitation speed was adjusted from 500 rpm initially to 400 rpm after 24 h. The *dhaD* gene using the T7 promoter in the pETM6 plasmid was overexpressed by addition of IPTG after 6 h as an inducer. [↑](#footnote-ref-3)
